# Self-adjuvanted RNActive^® ^vaccine induces local immune responses at the injection site leading to potent adaptive immunity in mice and humans

**DOI:** 10.1186/2051-1426-2-S3-P172

**Published:** 2014-11-06

**Authors:** Aleksandra Kowalczyk, Fatma Döner, Edith Jasny, Janine Noth, Birgit Scheel, Sven D Koch, Mariola Fotin-Mleczek, Regina Heidenreich

**Affiliations:** 1 CureVac GmbH, Tuebingen, Germany

## 

Two-component mRNA-based vaccine (RNActive^®^) combines high antigen expression with strong immune stimulation. We have previously shown that intradermal administration of RNActive^® ^vaccines induced balanced, potent and long-lasting immune responses in both mice and humans. Here, we characterized in detail early events after vaccine injection to better understand the self-adjuvant capacity of mRNA-based vaccines. Shortly after intradermal administration, mRNA was present in both non-leukocytic and leukocytic cells, the latter being mostly represented by MHC class II-expressing cells. Further studies revealed that 24 h after intradermal administration RNActive^® ^was detectable in dendritic cells (DCs) within the draining lymph nodes (dLNs). Full genome microarray analyses of the skin tissues showed that RNActive^® ^vaccine transiently altered the gene expression profile at the injection site. Various chemokines such as CXCR3 ligands (including CXCL9, CXCL10 and CXCL11) as well as CCL2, CCL4, CCL5 and CCL12, whose pleiotropic functions include recruitment and activation of macrophages, NK cells and DCs, were up-regulated early after RNActive^® ^treatment. Additionally, gene expression of multiple proinflammatory cytokines such as IL-6 or TNF-α was increased. In corroboration with the microarray data, after injection of mRNA vaccine we observed a strong production of chemokines including CXCL9, CXCL10, CCL3, CCL4, CCL5 as well as proinflamatory cytokines (IL-6, TNF-a) at the site of injection. No changes in the cytokine serum level were detected suggesting a local immunostimulation with no signs of cytokine release syndrome. The innate immune responses in the skin were followed by the increased cellularity as well as an enhanced activation of a wide range of the immune cells in the dLNs including CD8^+ ^T-cells, B cells, γδ T-cells, NK and NKT cells. In summary, our data indicate that the self-adjuvanted RNActive^® ^vaccine induces strong innate immune response at the site of administration followed by the activation of the immune cells in the dLNs. These results provide a possible mechanistic explanation of a potent and balanced adaptive immunity induced by mRNA-based vaccine which is a novel vaccination platform for an efficient cancer immunotherapy.

